# Long-term, medium-term and acute stress response of urban populations of Eurasian red squirrels affected by different levels of human disturbance

**DOI:** 10.1371/journal.pone.0302933

**Published:** 2024-05-03

**Authors:** Agata Beliniak, Jakub Gryz, Daniel Klich, Rafał Łopucki, Ilona Sadok, Kinga Ożga, Karolina D. Jasińska, Agnieszka Ścibior, Dorota Gołębiowska, Dagny Krauze-Gryz

**Affiliations:** 1 Department of Forest Zoology and Wildlife Management, Warsaw University of Life Sciences, Warsaw, Poland; 2 Department of Forest Ecology, Forest Research Institute, Sękocin Stary, Raszyn, Poland; 3 Department of Animal Genetics and Conservation, Warsaw University of Life Sciences, Warsaw, Poland; 4 Department of Biomedicine and Environmental Research, The John Paul II Catholic University of Lublin, Lublin, Poland; 5 Department of Chemistry, Faculty of Medicine, The John Paul II Catholic University of Lublin, Lublin, Poland; University of Veterinary Medicine Vienna: Veterinarmedizinische Universitat Wien, AUSTRIA

## Abstract

Animals in urban areas often encounter novel and potentially stressful conditions. It is important to understand how wildlife cope with anthropogenic disturbance. To investigate this specific adaptation we live-trapped squirrels in two study sites in Warsaw: a forest reserve and an urban park and we estimated stress responses at three levels: long-term and medium-term stress (the level of stress hormones, i.e. cortisol and cortisone concentrations, in hair and feces) and acute reaction to human-induced stress (measured during handling with the aid of the three indices: breath rate, struggle rate, and vocalization). According to GLMM models no difference in the stress hormones level was found between the two populations. The only differences in cortisol concentrations clearly depended on the season, i.e. being higher in autumn and winter comparying to other seasons. There was no influence of sex, or reproductive status on stress hormones. Forest squirrels had significantly higher breath rates, suggesting they were more stressed by handling. There was no difference in the struggle rate between study areas, this index was mostly affected by season (i.e. being highest in winter). First-trapped squirrels vocalized less than during the subsequent trappings. Assumingly, during the first, and more stressful trapping, squirrels used ‘freezing’ and/or little vocalization, while during next captures they used alarm calls to warn conspecifics. Overall, we showed that the two squirrel populations differed only in terms of their breath rate. This suggests that they did not differ in medium-term and long-term stress in general, but they can differ in acute response to handling. This also suggests that both populations were similarly affected by environmental factors. The lack of clear effects may also be due to population heterogeneity. Thus, in order to assess the effects of anthropogenic stressors a broader range of indicators and diverse analytical methods, including behavioral analyses, should be employed.

## Introduction

Urbanization is known to be one of the most drastic environmental changes caused by humans [1]. Animals living in urban areas often encounter novel conditions, which may be potentially stressful, like changes in predation pressure, altered food resources, and new species interactions [2] or increased disturbance from people [3]. However, some animals can adapt and survive or even thrive in cities [4–7]. Since cities and their infrastructure are rapidly expanding [4], it is increasingly important to understand how wildlife cope with anthropogenic disturbance [8].

Squirrels are one of the typical mammalian groups which have colonized urban areas and adapted to them successfully. This was observed for the Eurasian red squirrel (*Sciurus vulgaris*) [9–11], the grey squirrel (*S*. *carolinensis*) [12] and the fox squirrel (*S*. *niger*) [13]. Squirrels in urban habitats may exhibit higher population densities [9,12,14], higher aggressiveness [12], increased boldness [15] and different activity patterns than in a non-urban environment [16,17]. Red squirrels are commonly fed by people in parks and gardens [18–24] and may be considered an iconic species of coexistence between human and wildlife in urban areas [25].

Levels of glucocorticoid hormones, such as cortisol or corticosterone, provide information about the impact of environmental factors on wildlife [26,27]. Measuring hair cortisol and fecal cortisol metabolites as an indicator of adrenocortical activity in animals provides an insight into stress responses of animals over medium-term and long-term time scales, thus being a valuable tool in environmental studies [28,29]. Anthropogenic disturbances can be assumed as stressors to wildlife, and as a result, animals in contact with urban areas and humans are expected to have higher glucocorticoid levels compared to animals that are less exposed or not exposed at all [30–32]. It is often assumed that urban populations show higher baseline levels of glucocorticoid hormones because of chronic stress, however, results are often inconsistent (reviewed in Iglesias-Carrasco *et al*. [8]). It has also been suggested that animals living in urban habitats show reduction of HPA sensitivity through habituation [33], which may help animals to adapt to novel environments.

The analysis of hair and fecal glucocorticoid metabolites is considered less invasive compared to approaches that include blood sampling. For this reason, it has been applied to many wildlife species to monitor their stress responses to various disturbances [8,29,34,35]. Glucocorticoids concentrations in squirrels can depend on different factors like population density [36,37], sex [38–42], season [38,41], body mass [38,40] and condition [37,43]. To date there has been little agreement on how much urbanization influences stress levels in wildlife. In one study it was noted that levels of fecal cortisol metabolites were highest in areas where human disturbance was greatest [36]. On the other hand, chipmunks (*Tamias striatus*) inhabiting urban areas had lower concentrations of fecal cortisol than those from natural habitats [39]. The opposite effect was reported in a recent study in Japan, where levels of the fecal cortisol metabolites were not significantly different between the urban and rural squirrel populations [42,44].

In order to estimate acute stress responses to direct anthropogenic stressors, mainly behavioural indicators are used, such as: breath rate, struggle rate, and vocalisation, which show the response to handling [45–48]. Breath rate (i.e the frequency of respiratory acts) of an animal is assumed to be an index of the emotional and the stress response, similar to cardiovascular parameters, such as heart rate [46,49]. On the other hand, struggle rate, which is the amount of time animal moves around the bag after trapping, can be a measure of docility [50] and has been shown to correlate with aggressiveness and represent boldness [45–46]. Finally, the vocalization while handling can be measured. Squirrels use vocal communication to transfer vital information between conspecifics [51], which could be more difficult to obtain by using visual communication [52]. Tree squirrels produce different types of calls: alarm, agonistic, discomfort, mating, affiliative, and neonatal as well [51]. Alarm calls are the most frequently emitted and they typically serve as warning calls [53]. A call-type might also have one or several behavioral functions [51]. Here we assumed that vocalization during handling would show a reaction to potential danger and serve as an alarm call.

Behavioral traits may have fitness consequences [45,54] and intra-specific variation in capacity to cope with environmental challenges may buffer the species for strong fluctuations in the natural habitat [55]. Highly aggressive individuals adopt a proactive coping style whereas low levels of aggression indicate a more passive or reactive style of coping [55]. The two coping styles might explain a differential vulnerability to negative influence of stress due to the differential adaptive value of the two coping styles and the accompanying neuroendocrine differentiation [56]. A proactive coping animal may be adapted to stable environmental conditions. The reactive coping style may do better under variable and unpredictable environmental conditions [57]. Thus, they can determine how animals cope with environmental changes such as urbanization [58]. Therefore, in this study, the stress response of two urban red squirrel populations, which inhabit two different areas: an urban park and an urban forest reserve, both placed within city districts, was compared. The park squirrels live at very high densities [14], receiving plentiful supplementary food from park visitors [20,22], whereas the effect of human disturbance on the forest population should be considered negligible. We attempted to estimate stress responses at three levels: long-term and medium-term stress, revealed by the level of cortisol and cortisone concentrations in hair and feces, and acute reaction to human-induced stress, measured with the aid of the three indices: breath rate, struggle rate, and vocalization. We aimed to find out if squirrel behavior in respone to handling changed when they gained experience through multiple captures. We also hypothesized that in the urban forest, where squirrels live in lower density and generally maintain better body condition [14], the animals would exhibit lower concentrations of stress hormones. On the other hand, we supposed that the response to handling (vocalization, breath, and struggle rate) in the urban park (a site with higher human disturbance) would be less noticeable as park squirrels have direct and frequent encounters with humans [22,59].

## Material and methods

### Study area

We trapped squirrels in two study sites, both located in Warsaw, the capital of Poland. Warsaw is located in the central part of the country and has approximately two million inhabitants. This region is affected by both dry continental and harsh climates of Eastern Europe and Asia and the mild oceanic climate of Western Europe. Mean ambient temperature ranges from -4°C in January to 18°C in July. However, minimum temperature may be below -30°C and the maximum temperature may rise above 35°C.

One of the study sites was located in Royal Łazienki Museum, in a park in the central district of the city (hereafter ‘urban park’). The park and its architectural attractions are very popular among visitors and local inhabitants. This 76-hectare green area is surrounded by busy streets and built-up areas. More than 90 species of trees and shrubs of both native and foreign species grow in the park [9]. About 20% of trees reach more than 150 years [9]. Tree species are mostly deciduous, e.g. common oak (*Quercus robur*), common beech (*Fagus sylvatica*), common hornbeam (*Carpinus betulus*), common hazel (*Corylus avellana*), English walnut (*Juglans regia*) and North American walnut (*Juglans nigra*). Apart from the natural food base, animals (birds and squirrels) are also fed by both park employees and visitors [14]. The most common supplemental food includes different kinds of seeds, e.g. sunflower (*Helliantus annuus*), hazelnut, and walnut [20,22].

The other study site was Natolin Forest Reserve (hereafter ‘urban forest’), a forest located approximately 10 km from the city center. This area, which served as a royal hunting ground in the seventeenth century, was turned into a parkland that extended around the residences of Polish magnates in the eighteenth century. After the Second World War, the area was used as a government residence. Spontaneous regenerations of woodland occurred during the post-war period, nowadays the whole area of the reserve area is tree-covered. The reserve covers 105 ha and has been protected since 1991. It is closed to the public and permission is needed for an entrance. The oldest stands are more than 250 years old, dead or fallen trees are left for natural decomposition and only natural regeneration occurs. There are built-up areas to the west of the reserve, whereas on the other side it is surrounded by farmland. Trees are mostly deciduous like common hornbeam, common oak, ash (*Fraxinus excelsior*), elms (*Ulmus* spp.), common hazel, and black alder (*Alnus glutinosa*).

The two areas are approximately 10 km from each other and potentially interconnected via the the Warsaw Escarpment, which spans along the Warsaw section of the Vistula River. Nevertheless, this is intersected by roads and buildings so ecological connectivity between the two areas is highly disturbed. The urban park is much more affected by the urban heat island effect than the urban forest [60]. The two squirrel populations vary in density: the MNA (minimum number alive) value obtained in previous sudy for the urban park squirrels ranged from 1.05 to 1.89 ind./ha and from 0.2 to 0.28 ind./ha for forest squirrels. Adult red squirrels in the forest had higher body masses than those in the urban park (355 g and 337g respectivelly) [14]. Red squirrels in the forest had also significantly better conditions but were less sexulally active than these in the urban park (see details in Beliniak *et al*. [14]).

### Live-trapping

Access to the Natolin Forest Reserve and red squirrel capture was allowed with permission issued by the General and Regional Directorates for Environmental Protection (WPN-I.6205.124.2018.AS and WPN-I.6401.208.2018.PF). Trapping and handling squirrels complied with current laws on animal research in Poland and was carried out with a permit from Local Ethical Committee (WAW2/072/2018).

Our study lasted from July 2018 to December 2020. We used standard wire mesh live traps (51×15×15 cm) (manufactured by “Jerzyk” Jerzy Chilecki, Białowieża, Poland). We live-trapped squirrels with 30 traps in the urban park and 40 traps in the urban forest. The traps were located on the ground or in trees on wooden platforms and were partly covered by black plastic to provide shelter from rain and snow. Before trapping, traps were pre-baited with hazelnuts and English walnut for seven days. After that we conducted trapping session: we baited and set traps for four (in most cases) to nine days. Trapping was conducted in both areas in the same month, in total during thirteen trapping sessions (i.e. in 2018: July, September, and November; in 2019: January, March, May, July, September; in 2020: March, May, July, October, December). Traps were set in the morning (around 6–7 a.m.), checked after 2–4 hours, and secured for the night (in a manner, which prevented them from being closed). Every trapped squirrel was flushed into a light hessian bag then we recorded the struggle rate for 30 sec.–the duration of time a squirrel kept moving in the bag. Then, to minimalize stress during handling, a squirrel was flushed into a wire mesh handling cone [61] and the breath rate estimated, i.e. the chest moves were counted during the 20 sec. Each newly trapped squirrel was marked with an individually numbered ear-tag (2x8 mm, National Tag&Band, Newport, KY, USA), weighted to the nearest 10 g (Pesola spring balance) and measured with tapmeasure right hind foot (without claws). Subsequently we defined sex and reproductive status of squirrels. Females were determined as non-breeding (anoestrous, small vulva, no longitudinal opening), or breeding. The latter category included: postoestrous and pregnant (swollen vulva with longitudinal opening, enlarged belly during pregnancy) or lactating (large nipples, milk excretion could be stimulated). Males were recorded as non-breeding (abdominal testes or semi-scrotal and scrotum small) or breeding (testes scrotal and scrotum large) [62]. We defined if a squirrel was sub-adult or adult. Sub-adult females had a very small vulva and the nipples were still invisible, males had small scrotum and abdominal testes. Older animals were considered adult [63]. Vocalization of squirrels was defined and ordered in increasing intensity from 1 to 4: 1 –none, 2 –growling, 3 –yelling, 4– screaming. At least two (usually three) people were involved in the trapping to make estimates as precise as possible. After handling we collected fresh feces left in the traps (not contaminated by the urine) to estimate fecal glucocorticoid metabolite levels. In Eurasian red squirrels, the fecal glucocorticoid metabolite levels from initial capture do not significantly differ from the hormone levels of at least 48 hours after the capture event [38]. Therefore, samples collected from recaptured squirrels were also analyzed if they were at least 48 hours apart. Fecal samples were collected into 2 ml centrifugal tubes, maximum 4 hours after setting a trap, immediately cooled in ice, and transported to the laboratory, where they were stored in a freezer at −20°C until analysis. Additionally, during handling, a small hair sample (up to 50 mg) was collected. The hair was always sampled from the same region of the body (the middle part of the tail) and was cut as close as possible to the skin using fine scissors. Samples were stored in string plastic bags in the -24°C freezer until analysis.

### Laboratory analysis

#### Determination of hair steroids (cortisol and cortisone)

In hair samples, cortisol and its metabolite—cortisone were determined using a validated ultra-high performance liquid chromatography coupled to electrospray ionization-tandem mass spectrometry (UHPLC-ESI-MS/MS) method. We decided to follow the concentration of two steroids in the hair because it has beed suggested that measuring multiple glucocorticoids simultaneously may provide more comprehensive information and more reliable interpretation of the acquired results [34]. The method of sample preparation and analysis was described in detail by Sadok *et al*. [34]. Briefly, hair samples were washed twice with isopropanol, dried overnight, cut into small pieces into a tube in an amount from 16 to 48 mg, and milled using steel beads. The steroids were extracted in the dark with 1 ml of methanol (LC-MS grade) fortified with internal standard (10 μg/ml of cortisol-D_4_) for 24 hours. The supernatants were further purified by solid-phase extraction using the polymeric reversed phase Strata-X cartridges (30 mg/1 ml) supplied from Phenomenex as described previously (Sadok *et al*. [34]).

**Fig 1:**
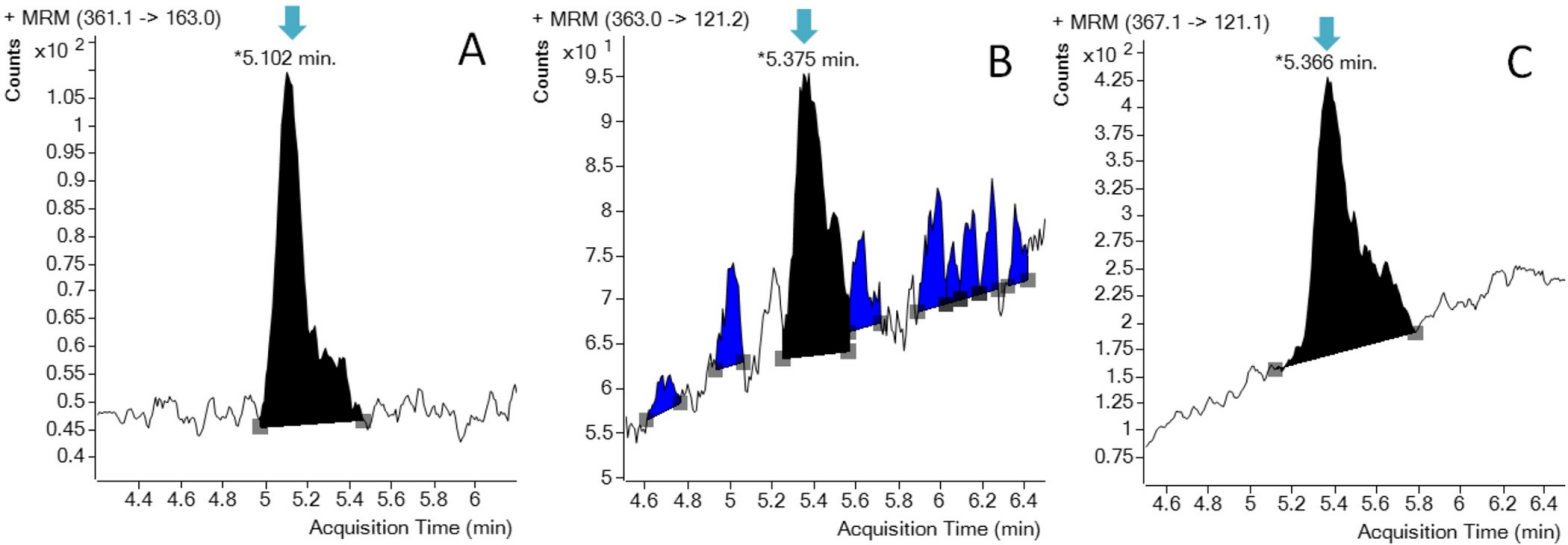
An example of UHPLC-ESI-MS/MS data obtained during the analysis of squirrel hair sample. Arrows indicate the position of signals of cortisone (A), cortisol (B) and cortisol-D4 used as isotopically labeled internal standard (C).

Samples were analyzed using a 1290 infinity UHPLC system coupled to a 6460 triple quadrupole mass spectrometer equipped with the Jet Stream electrospray ion (ESI) source from Agilent Technologies (Santa-Clara, CA, USA) and settings detailed in Sadok *et al*. [34]. The concentrations of steroids were determined in μg/ml from matrix-based calibration plots after signals normalization on the internal standard. The final results were expressed in pg/mg after conversion to the weight of hair samples subjected to extraction. An example of data obtained during the UHPLC-ESI-MS/MS analysis showing the presence of hair cortisol and cortisone in squirrel hair sample is presented in Fig 1.

#### Determination of fecal cortisol

The concentration of cortisol in the fecal samples was assessed with the ELISA method using a commercial kit with antibodies for this hormone (COR ELISA Kit No. EU0391, Wuhan Fine Biological Technology Co.). The analysis was performed according to the manufacturer’s protocol described in detail previously [64,65] as follows: 100 mg samples of feces were weighed using an XA 100 3Y analytical balance (Radwag, Poland), mixed with 0.5 ml of Dulbecco’s phosphate-buffered saline (DPBS, Thermo Fisher Scientific) without calcium and magnesium (pH = 7.0–7.3) in a centrifugal tube and shaken for 10 min using multivortex. Next, the suspension was centrifuged at room temperature (at 10000 rpm for 20 min) using a Heraeus Megafuge 11R centrifuge (Thermo Fisher Scientific, Germany). The obtained supernatants were immediately used for analysis using a Synergy 2 multi-mode microplate reader (BioTek Instruments, Inc. USA) equipped with an automated microplate strip washer (ELx50, BioTek Instruments, Inc. USA) and an ELMI DTS-4 digital thermostatic microplate shaker (Riga, Latvia). The concentration of cortisol in the samples was determined by comparing the optical density (OD) of the samples to the standard curve, the range of which was from 0.39 to 25 ng/ml. A separate calibration curve was made for each plate using eight different standard dilutions (0, 0.391, 0.781, 1.562, 3.125, 6.25, 12.5 and 25 ng/ml). Each dilution was duplicated. Intra- and inter-assay coefficients of variation were 10.6% and 12.8%, respectively. Samples with a concentration of cortisol above the upper limit of the curve were diluted and reassayed. We did not have any samples with a cortisol concentration below the lower range of the curve. The OD values of the samples were measured at 450 nm at room temperature. As in papers of Klich *et al*. [64,65], the concentrations of fecal cortisol were normalized per weight of feces and finally expressed as nanograms of cortisol per one gram of dry mass of feces (ng/g). We additionally used cortisone and corticosterone standards (Sigma-Aldrich, USA) at concentrations of 5 and 15 ng/ml to confirm the selectivity of the used ELISA kit (no reaction was observed).

### Statistical analysis

To verify if the stress variables differentiate between the urban park and the urban forest squirrels, six generalized linear mixed models were run, of which three models with dependent variables related to long-term and medium-term stress: 1) hair cortisol concentration, 2) hair cortisone concentration, 3) fecal cortisol concentration, and three models with dependent variables indicated acute stress: 4) breath rate, 5) struggle rate, and 6) vocalization. There were six explanatory variables in each model, i.e.: SITE, SEASON, AGE, EXPERIENCE, REPRODUCTIVE STATUS, and CONDITION. SITE was a grouping variable of the two study sites: urban park (Łazienki) and urban forest (Natolin). SEASON was a grouping variable of four astronomical seasons: spring (1 March–31 May), summer (1 June–31 August), autumn (1 September–30 November), and winter (1 December–28 February). For hair hormone concentration winter was not included due to lack of hair samples from this period. AGE was a grouping variable of two age groups: adults and subadults. REPRODUCTIVE STATUS was a grouping variable dependent on sex, we distinguished two activity types for each sex: non-breeding males, non-breeding females, breeding males and breeding females. EXPERIENCE was a grouping variable of two squirrel groups: a) re-trapped–the squirrels which had been trapped before, and b) first-trapped–squirrels trapped for the first time. CONDITION was a covariate calculated based on residuals of log body mass and foot length (see: [14]). For Hair cortisol, Hair cortisone, Fecal cortisol concentrations and Struggle rate we used gamma distribution with log link function, for Breath rate and Vocalization we used negative binomial distribution with log link function. The IDs of squirrels were set as a random effect in the models to account for the repeated sampling of individual animals. Restricted maximum likelihood (REML) was used to estimate the parameters in the best model obtained. Model selection was based on the corrected Akaike information criterion (AICc) values in a multi-model selection procedure [66]. All possible model permutations were performed and, finally, the models were ranked according to their Akaike weights (ωi). The principle of model selection was lower AIC values. In total, a maximum of 336 red squirrel individuals were tested, however, due to limitations in the possibility of collecting material, the number of analyzed observations differed. For each model, the sample size was as follows: n = 93 for hair cortisol and cortisone concentrations, n = 112 for fecal cortisol concentration, n = 333 for breath rate, n = 336 for struggle rate, and n = 335 for vocalization. In general, twice as many individuals were examined in urban park as in forest and there were twice as many re-trapped squirrels as first-trapped (Table 1). All statistical analyses were performed with IBM SPSS v29.0 (Armonk, New York).

**Table 1:** Number of samples from squirrels first-trapped (FIRST) and re-trapped (RET) in a given study site.

Indicator	Urban forest		Urban park		Total
	FIRST	RET	FIRST	RET	
Hair cortisol concentration	11	20	22	40	93
Hair cortisone concentration	11	20	22	40	93
Fecal cortisol concentration	12	23	16	61	112
Breath rate	28	78	82	139	333
Struggle rate	28	86	82	140	336
Vocalization	28	85	89	133	335

## Results

Squirrels from urban forest presented lower levels of hair cortisol concentration, fecal cortisol concentration than squirrels front urban park. However, similar levels of hair cortisone concentration, struggle rate ans vocalization was observed. Slightly higher breath rate in forest squirrels was observed comparing to urban squirrels (Table 2).

**Table 2:** Mean (±SE) levels of stress indicators observed in forest and urban squirrels (detailed data is presented in S1 Table).

Indicator	Urban forest		Urban park	
	Mean	SE	Mean	SE
Hair cortisol concentration [ug/g]	0.033	0.005	0.047	0.005
Hair cortisone concentration [ug/g]	0.046	0.003	0.048	0.002
Fecal cortisol concentration [ng/g]	29.209	4.603	62.976	8.483
Breath rate [chest moves/20 sec.]	28.661	0.451	25.597	0.248
Struggle rate [body moving time/30 sec.]	7.178	0.481	7.956	0.400
Vocalization [rank: 1–4]	1.619	0.086	1.820	0.076

### Hair cortisol concentration

**Fig 2:**
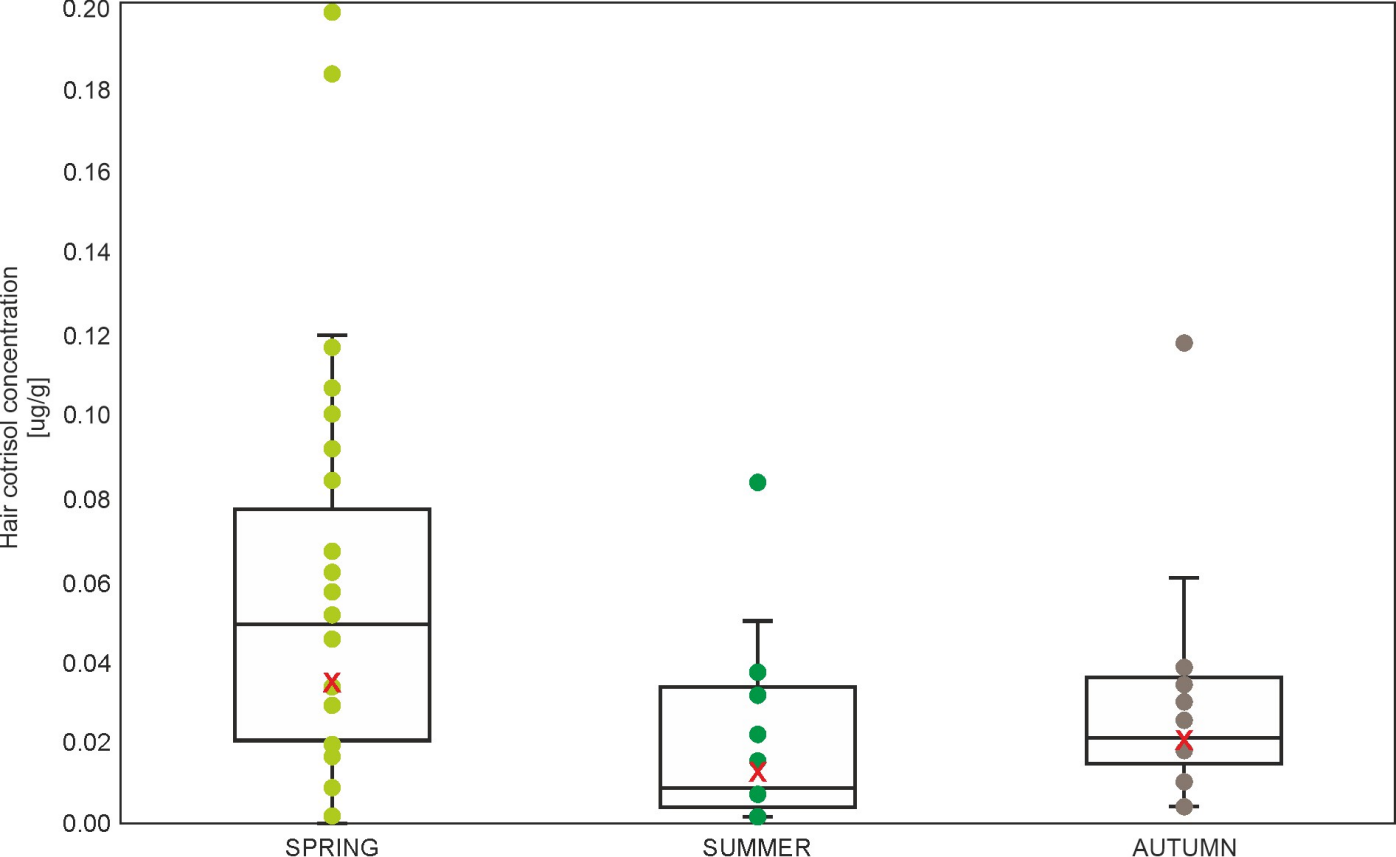
Boxplot with individual data points and mean (red X) hair cortisol concentration in squirrels with regard to SEASON (marginal means from generalized linear mixed model, S3 Table).

The highest ranked model contained AGE, SEASON, and CONDITION, but only season showed a statistically significant effect (Table 3). Squirrels presented higher hair cortisol concentration during spring, but lower during summer compared to the hair cortisol concentration in autumn (Table 3, Fig 2). CONDITION showed a nonsignificant, positive effect, squirrels with better condition presented higher levels of fecal cortisol concentration (β = 1.418, p = 0.458). AGE showed also nonsignificant effect, where adult squirrels presented higher hair cortisol concentration (β = 0.505, p = 0.216). Other variables were excluded during the model selection, but within the ΔAICc = 2 there were five models, which also included SITE and EXPERIENCE. None of these models included REPRODUCTIVE STATUS (S2 Table).

**Table 3:** Effect of AGE, SEASON, and CONDITION on hair cortisol concentration in squirrels in the highest ranked generalized linear mixed model (0*–reference category), REPRODUCTIVE STATUS, SITE, and EXPERIENCE were excluded during model selection (marginal R^2^ = 0.144 and conditional R^2^ = 0.690 for the highest ranked model).

Source	Beta	SE	t	p	Lower CI	Upper CI
Intercept	-3.454	0.8530	-5.323	<0.001	-6.236	-2.845
AGE (adult)	0.505	0.4051	1.246	0.216	-0.300	1.310
AGE (sub-adult)	0*					
SEASON (summer)	-0.489	0.2370	-2.064	0.042	-0.960	-0.018
SEASON (spring)	0.520	0.1875	2.775	0.007	0.148	0.893
SEASON (autumn)	0*					
CONDITION	1.418	1.9009	0.746	0.458	-2.360	5.195

### Hair cortisone concentration

**Fig 3:**
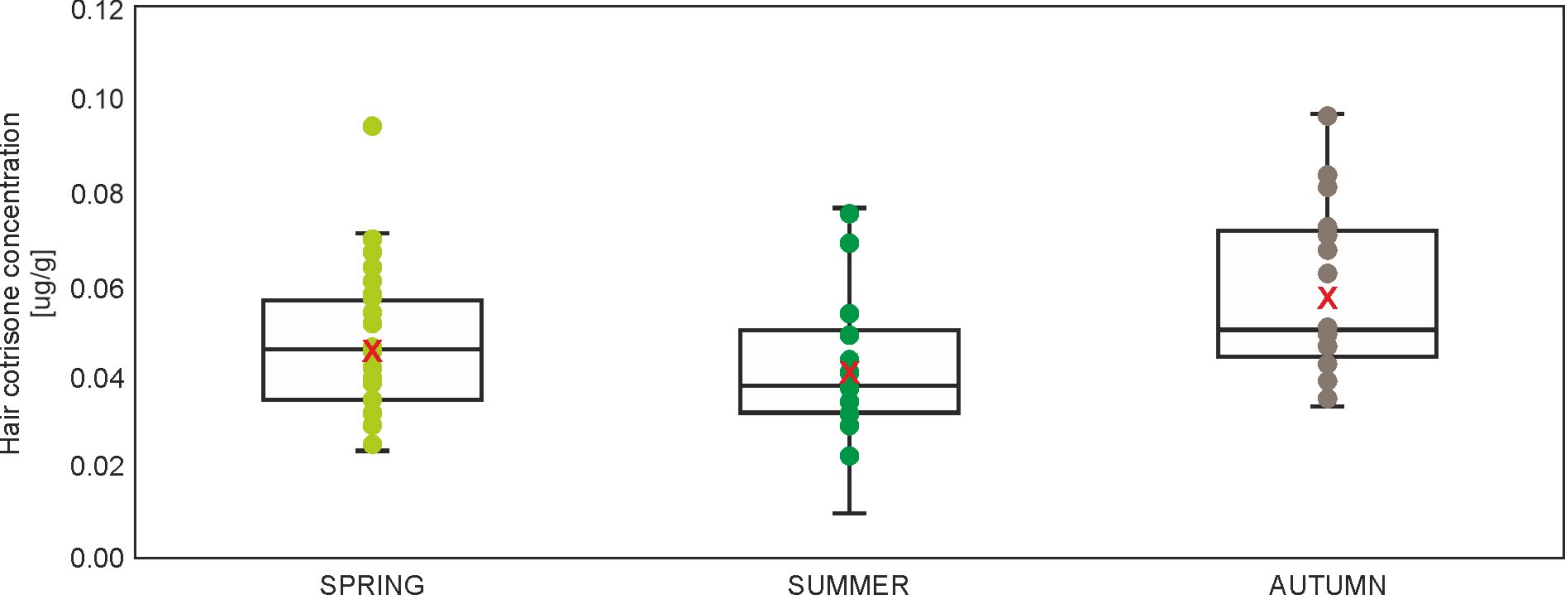
Boxplot with individual data points and mean (red X) hair cortisone concentration in squirrels with regard to SEASON (marginal means from generalized linear mixed model, S3 Table).

Only SEASON showed a statistically significant effect on hair cortisone concentration in squirrels, although also CONDITION was included in the model (Table 4). Squirrels in spring and summer presented lower hair cortisone concentrations than in autumn (Table 4, Fig 3). CONDITION showed nonsignificant, negative effect on hair cortisone concentration (β = -0.482, p = 0.367) (Table 4). Other variables were excluded during the model selection, but within the ΔAICc = 2 there were three models, which also included AGE (S2 Table).

**Table 4:** Effect of SEASON and CONDITION on hair cortisone concentration in squirrels in the highest ranked generalized linear mixed model (0*–reference category), REPRODUCTIVE STATUS, AGE, SITE, and EXPERIENCE were excluded during model selection (marginal R^2^ = 0.074 and conditional R^2^ = 0.480 for the highest ranked model).

Source	Beta	SE	t	p	Lower CI	Upper CI
Intercept	-2.742	0.3022	-9.075	<0.001	-3.343	-2.142
SEASON (summer)	-0.331	0.0997	-3.321	0.001	-0.529	-0.133
SEASON (spring)	-0.214	0.0807	-2.650	0.010	-0.374	0.053
SEASON (autumn)	0*					
CONDITION	-0.482	0.5315	-0.907	0.367	-1.538	0.574

### Fecal cortisol concentration

**Fig 4:**
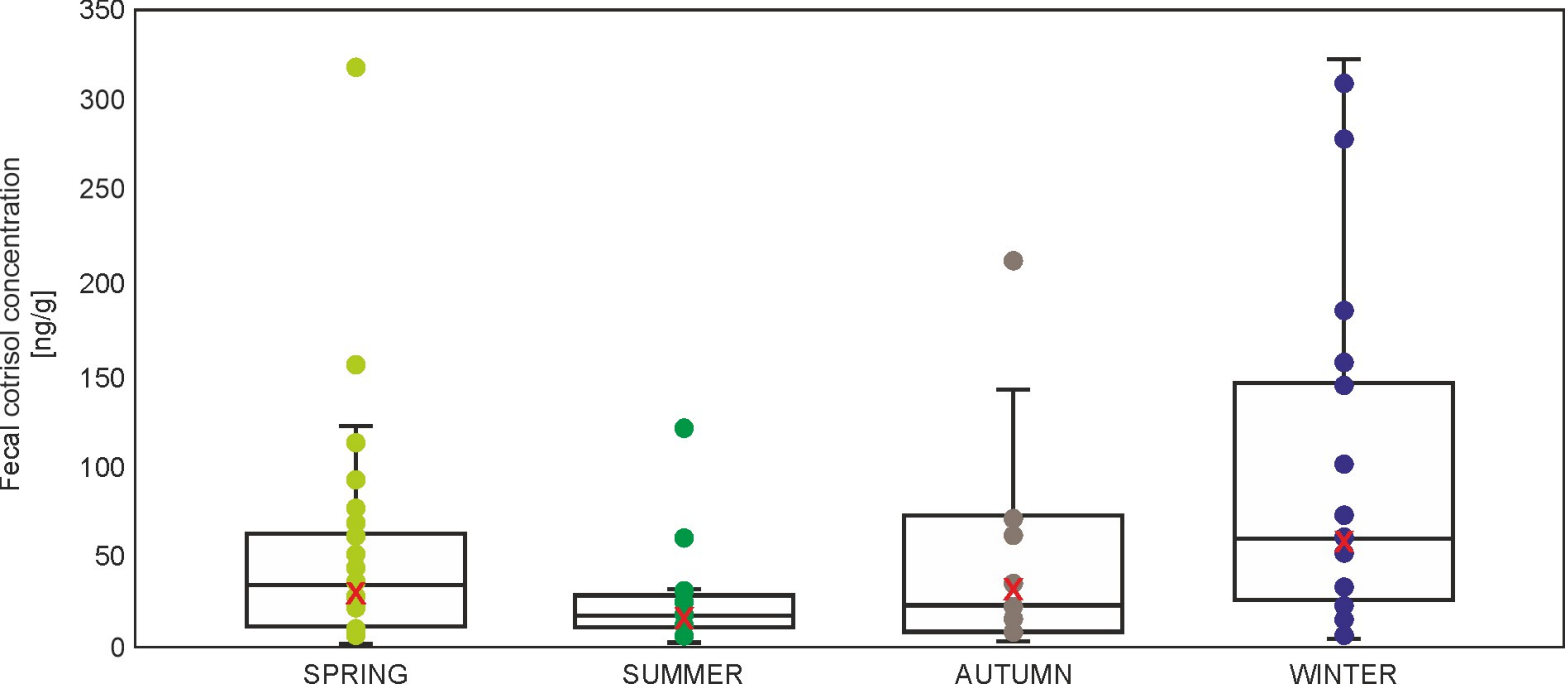
Boxplot with individual data points and mean (red X) fecal cortisol concentration in squirrels with regard to SEASON (marginal means from generalized linear mixed model, S3 Table).

The highest ranked model contained only SEASON and CONDITION. Nevertheless, within ΔAICc = 2 there were eight models which included also SITE, AGE, and EXPERIENCE. The highest ranked model presented much lower AICc values than the null model (ΔAICc = 18.6) (S2 Table). Cortisol presented significantly lower values in spring and summer compared to winter (Table 5, Fig 4). CONDITION showed nonsignificant, positive effect on fecal cortisol concentration (β = 0.583, p = 0.737) (Table 5).

**Table 5:** Effect of SEASON and CONDITION on fecal cortisol concentration in squirrels in the highest ranked generalized linear mixed model (0*–reference category), REPRODUCTIVE STATUS, AGE, SITE, and EXPERIENCE were excluded during model selection (marginal R^2^ = 0.081 and conditional R^2^ = 0.653 for the highest ranked model).

Source	Beta	SE	t	p	Lower CI	Upper CI
Intercept	3.906	1.0048	3.888	<0.001	1.914	5.898
SEASON (autumn)	-0.586	0.3135	-1.870	0.064	-1.208	0.035
SEASON (summer)	-1.266	0.2810	-4.504	<0.001	-1.823	-0.709
SEASON (spring)	-0.663	0.2191	-3.028	0.003	-1.098	-0.229
SEASON (winter)	0*					
CONDITION	0.583	1.7274	0.337	0.737	-2.842	4.007

### Breath rate

**Fig 5:**
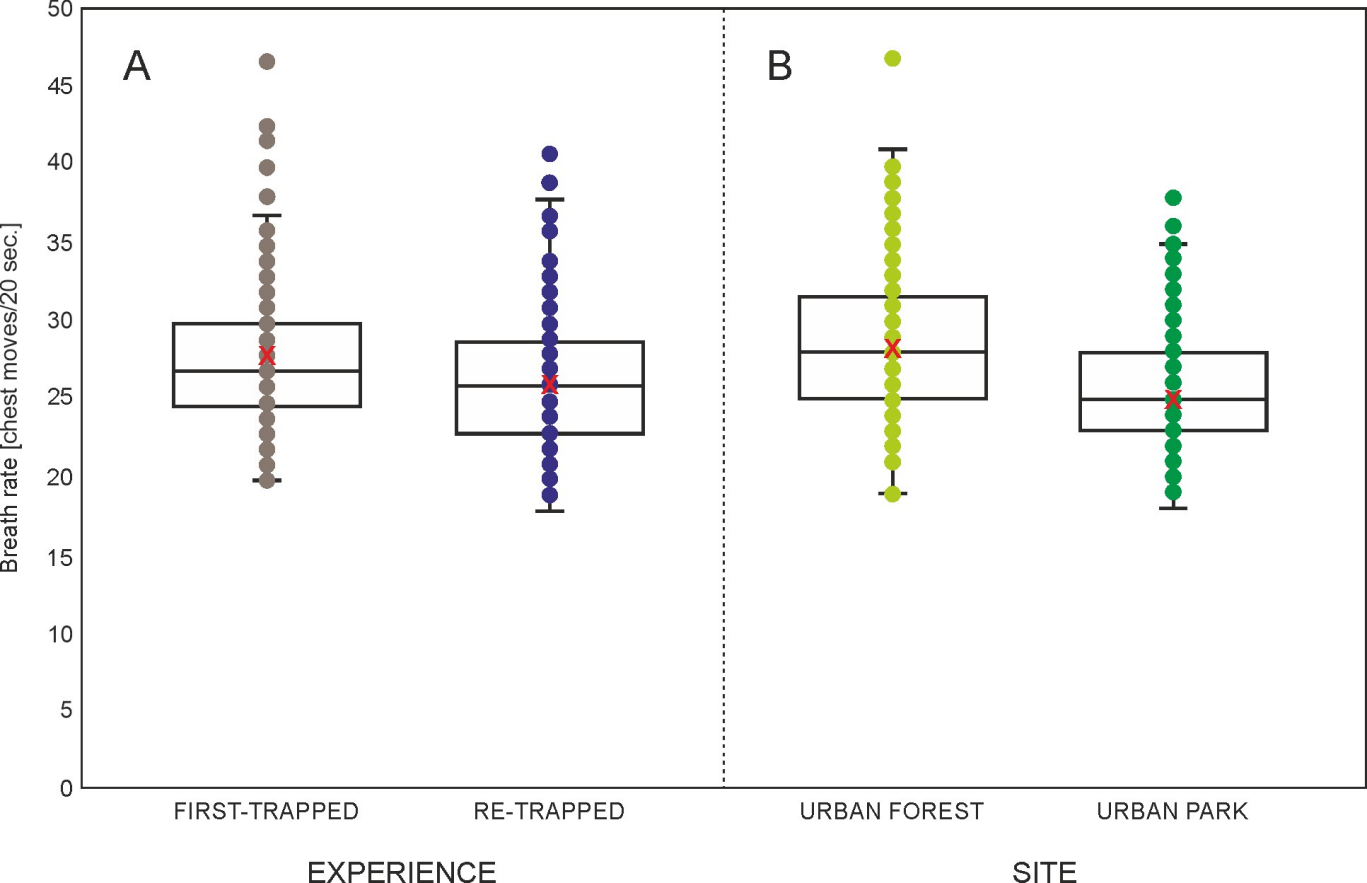
Boxplot with individual data points and mean (red X) breath rate in squirrels with regard to A) EXPERIENCE (first-trapped or retrapped) and B) SITE (urban park or urban forest) (marginal means from generalized linear mixed model, S3 Table).

For the breath rate the highest ranked model included SITE, EXPERIENCE, and CONDITION. The remaining predictors were excluded during model selection. The highest ranked model’s AICc was lower only by 1.3 from the next ranked model with SITE and EXPERIENCE, but also 22.2 from the null model (S4 Table). Squirrels from the urban park (Łazienki) presented a lower breath rate (β = -0.112, p < 0.001) than squirrels from the urban forest site (Natolin) (Table 6, Fig 5A). A higher breath rate (β = 0.067, p = 0.003) was also observed in first-trapped squirrels in comparison to re-trapped squirrels (Table 6, Fig 5B). The CONDITION effect is also noticeable, although not statistically significant (β = 0.334, p = 0.116). The squirrels showed a higher breath rate as their body condition level increased (Table 6).

**Table 6:** Effect of EXPERIENCE, SITE, and CONDITION on breath rate of squirrels in the highest ranked generalized linear mixed model (0*–reference category), REPRODUCTIVE STATUS, AGE, and SEASON were excluded during model selection (marginal R^2^ = 0.076 and conditional R^2^ = 0.274 for the highest ranked model).

Source	β	SE	t	p	Lower CI	Upper CI
Intercept	3.230	0.1251	25.806	0.000	2.983	3.476
EXPERIENCE (first-trapped)	0.067	0.0228	2.946	0.003	0.022	0.112
EXPERIENCE (re-trapped)	0*					
SITE (urban park)	-0.112	0.0230	-4.850	<0.001	-0.157	-0.066
SITE (urban forest)	0*					
CONDITION	0.334	0.2122	1.577	0.116	-0.083	0.752

### Struggle rate

**Fig 6:**
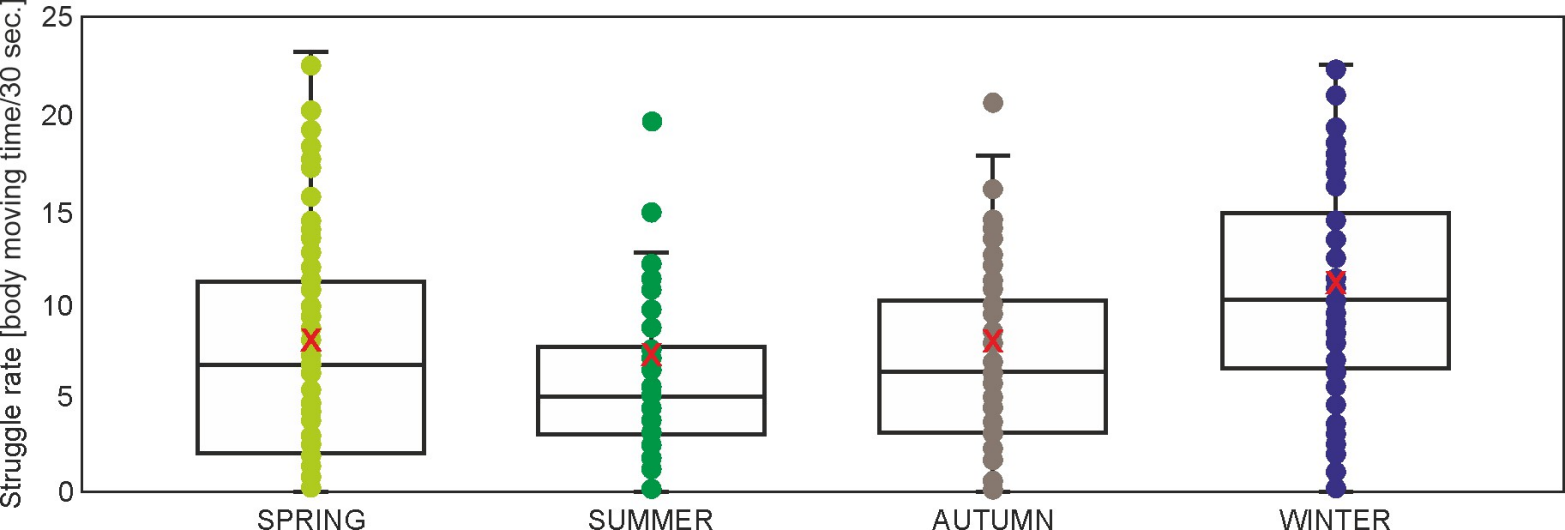
Boxplot with individual data points and mean (red X) struggle rate in squirrels with regard to SEASON (marginal means from generalized linear mixed model, S3 Table).

A less pronounced effect of analyzed variables was found in the case of the struggle rate. The highest ranked model included only SEASON and CONDITION, but within the ΔAICc = 2 there was also a model which included SEASON, CONDITION, and EXPERIENCE (S4 Table). Also, ΔAICc with null model was only 6 (S4 Table). The model effects indicated a significantly lower β coefficient for the struggle rate in squirrels in spring, summer, and autumn than in winter (Table 7, Fig 6). Similar to breath rate, the CONDITION effect was included in the model, and also not statistically significant (β = 667, p = 0.373). The squirrels showed a higher struggle rate as their body condition level increased (Table 7).

**Table 7:** Effect of SEASON and CONDITION on struggle rate of squirrels in the highest ranked generalized linear mixed model (0*–reference category), REPRODUCTIVE STATUS, AGE, SITE, and EXPERIENCE were excluded during model selection (marginal R^2^ = 0.037 and conditional R^2^ = 0.494 for the highest ranked model).

Source	β	SE	t	p	Lower CI	Upper CI
Intercept	2.217	0.6443	3.441	<0.001	0.949	3.484
SEASON (autumn)	-0.375	0.1106	-3.388	<0.001	-0.592	-0.157
SEASON (summer)	-0.485	0.1258	-3.858	<0.001	-0.733	-0.238
SEASON (spring)	-0.339	0.1018	-3.329	<0.001	-0.539	-0.139
SEASON (winter)	0*					
CONDITION	0.667	0.7483	0.892	0.373	-0.805	2.139

### Vocalization

**Fig 7:**
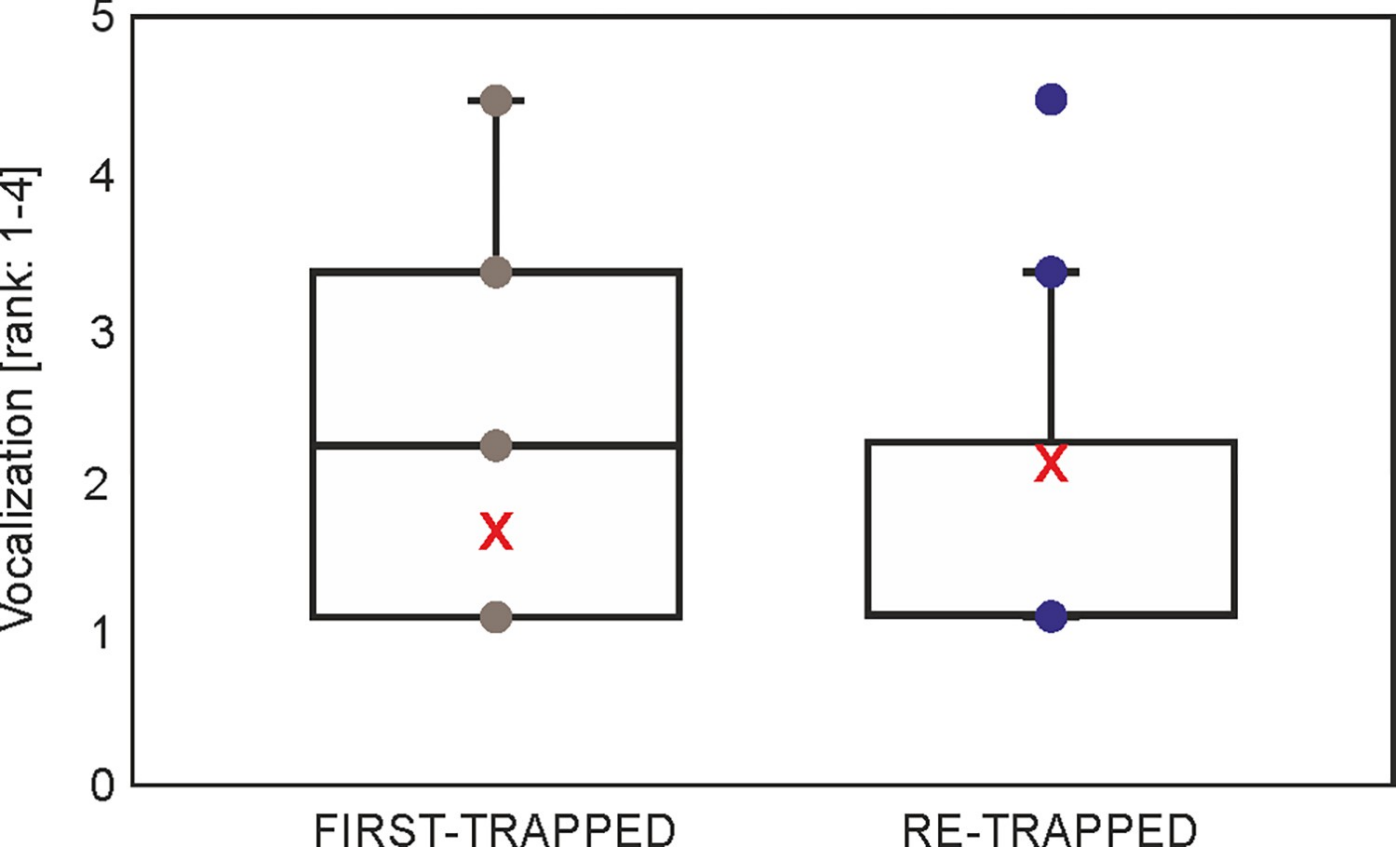
Boxplot with individual data points and mean (red X) vocalization in squirrels with regard to EXPERIENCE (first-trapped or retrapped) (marginal means from generalized linear mixed model, S3 Table).

Among the analyzed variables only EXPERIENCE and CONDITION had a weak effect on vocalization. Within ΔAICc = 2 there were five models, which, apart from CONDITION and EXPERIENCE, included also SITE and AGE (S4 Table). The highest ranked model had AICc lower by only 2.6 from the null model (S4 Table). Interestingly, the squirrel with no experience (i.e. first-trapped) presented lower vocalization than squirrels with the experience of being previously trapped (β = -0.201, p = 0.031) (Table 8, Fig 7). CONDITION also had a nonsignificant effect on the vocalization, but the opposite trend compared to previous models; i.e. squirrels with better condition presented a lower value of vocalization (β = -1.344, p = 0.069) (Table 8).

**Table 8:** Effect of EXPERIENCE and CONDITION on vocalization of squirrels in the highest ranked generalized linear mixed model (0*–reference category), REPRODUCTIVE STATUS, AGE, SITE, and SEASON were excluded during model selection (marginal R^2^ = 0.013 and conditional R^2^ = 0.185 for the highest ranked model).

Source	β	SE	t	p	Lower CI	Upper CI
Intercept	1.030	0.4390	2.347	0.019	0.167	1.894
EXPERIENCE (first-trapped)	-0.201	0.0929	-2.163	0.031	-0.384	-0.018
EXPERIENCE (re-trapped)	0*					
CONDITION	-1.344	0.7375	-1.822	0.069	-2.795	0.107

## Discussion

In this study, we focused on the response to stress of two red squirrel populations: a) to environmental stress (revealed by the level of stress hormones in hair and/or feces) and b) a reaction to acute human-induced stress (measured as a reaction to handling at a trapping event).

Contrary to our expectations, no pronounced site effect was found in long-term and medium-term stress. Herein, the only differences in cortisol and cortisone concentrations clearly depended on the season. This was confirmed by all three models for the concentrations of hormones (in hair and feces) (Tables 3–5, Figs 2–4). The squirrels in our study had higher levels of cortisol and cortisone concentrations in winter and autumn, which may be related to higher predation risk during foraging on the ground (higher visibility because of lack of leaves), reduced food quality, or more extreme weather conditions (snow and cold) [38].

### Stress hormones in hair and feces

The stress response of animals to various external factors (e.g., predation pressure) is a typical challenge for wild animals and results in a variety of adaptive responses [67]. However, urbanization poses a novel and previously unknown challenge for animals, and adaptation processes are likely occurring right before our eyes. Although it is generally assumed that urban populations should have higher levels of cortisol metabolite due to chronic stress [8], previous studies showed diverse results, i.e. higher levels of cortisol metabolites [68,69], lower levels [39,70,71] or no differences [42,72]. Variations among studies might be related to complex responses of neuroendocrine system to chronic stress or species/population/individual-dependent differences in the perception of stressors [8]. Our results are similar to a recent study conducted in Japan, which compared rural and urban populations of the Eurasian red squirrels [42]. Also, Martin and Réale [73] found that human frequentation did not affect cortisol levels in chipmunks, and suggested that human presence was not the main factor responsible for the stress reaction. If animals are exposed to a repeated non-lethal stressor, their stress response may decrease [74]. Indeed, in urban striped field mice *Apodemus agrarius* lowered concentrations of cortisol were observed, which suggested hormonal adjustment to urban conditions [71].

On one hand, urban habitats may be challenging to animals due to various disturbances, but on the other hand, they often offer greater availability of anthropogenic food and/or artificial feeding. According to some studies, cortisol concentration was shown to influence body condition [37,44] (or body mass [38,40,42]). Some others showed that higher body mass and better body condition may reduce glucocorticoid levels [75,76]. In our case, some effect on stress hormones can be also expected in relation to body condition, because in all three of the highest ranked models this explanatory variable was present (but nonsignificant). Interestingly, in our study, both hair and fecal cortisol concentrations increased with body condition. Squirrels’ body mass may be positively correlated with boldness and aggressiveness [77], which may explain this link between body mass and cortisol concentration.

Food abundance relates to physiological stress [19,78]. Supplementary food was already shown to make red squirrels shift their home ranges closer to supplementary food sources [23] and change their activity to adjust to human presence [16]. In our case, both study sites were deciduous and offered plentiful natural food sources but only park squirrels received anthropogenic food [14,20,22]. Yet, a much higher population density in the park might have resulted in higher intra-specific competition [14], which would wear off the positive effect of supplementary feeding in the park.

There was no influence of study site, sex, or reproductive status on stress hormones in all three of the highest ranked models, although these variables were incidentally present in some models within ΔAICc = 2, which indicates the possible effect of these variables on hormone levels. The not significant effect of sex reported in our case was in line with many studies [38,40,41,79,80]. However, the lack of a clear effect of reproductive status may be a result of masking the medium-term and long-term effect by individual variability and a clear influence of the season. It should be noted that during not only spring and summer, but also winter months squirrels are already engaged in mating chases and females may start lactating [81,82]. Indeed, sexually active squirrels were recorded in winter in our study, but also during spring and summer [14]. In previous studies the effects of the reproductive status of squirrels on the level of glucocorticoids levels were inconsistent. No effect as found in our case was in line with Santicchia *et al*. [40] but stands in contrast to other [38].

### Breath rate, struggle rate, and vocalization

We used three simple measures (breath rate, struggle rate, and vocalization) to compare squirrel reaction to acute human-induced stress between the study areas. As assumed, forest squirrels had significantly higher breath rates (suggesting they had stronger response to handling) than squirrels in a park. Urban squirrels are shown to adjust to human presence by altering their behavior, i.e. shortening the flight distance [15]. They become more tolerant to contact with humans, by staying on the ground and approaching people, which benefits squirrels with supplementary feed [20,22]. Also, in fox squirrels responses to human cues were reduced in urban as compared to less urban environments [83]. Increased respiration rate is one of the physiological components of stress, which shift metabolism toward energy mobilisation and away from energy conservation [84]. The breath rates were found to be indicators of acute stress in great tits [46]. We showed that first-trapped individuals were more stressed that the re-trapped ones, which points to the habituation (to a certain degree) of the stressor regardless of the study site.

Struggle rate can be interpreted as a part of behavioral stress response of an individual, but it is unknown if the stress response of an individual to handling (i.e. its magnitude) is correlated with the response to natural stress [85]. Nevertheless, the mean struggle rate of red squirrels was already shown to be higher in low density than in high density areas [47], while American red squirrels which struggled the most, also tended to be the most aggressive [45]. In our study, there was no difference in the struggle rate between forest and urban squirrels, this index was mostly affected by season (i.e. the struggle rate was highest in winter) but body condition (with a positive effect) was also included in the model. It is worth noting that the struggle rate is generally in line with the results of stress hormones, in which the seasonal effect is also pronounced. Glucocorticoids are hypothesized to serve as a major mechanism to cope with inclement weather [86]. It is thus possible that the seasonally elevated levels related to the struggle rate during handling, i.e. more pronounced activity of individuals in the face of imminent threat. It is worth noting, that a high inter-individual difference ocurred, indicated by relatively high conditional comparying to marginal R^2^ (Table 7). This may indicate different coping strategies in squirrels that mask the effects of the study site.

Alarm calls are vocalizations that alert other animals to impending danger [87]. These calls serve to warn conspecifics and signal the predator that its presence has been detected thus reducing chances of being preyed upon [87]. Here we measured the vocalization of squirrels as a response to handling (i.e. human-induced stress). Reactions of squirrels to some disturbance vary and range from ´freezing´ (remaining still) to alarm calling [18]. In our case, first-trapped squirrels vocalized less than during the subsequent trappings. Assumingly, during the first, and more stressful trapping, squirrels used ‘freezeing’ and/or little vocalization, and during next trapping they used alarm calls to warn conspecifics. Nevertheless, the explained variance of fixed and random effects was the lowest among all indicators (Table 8). It is still questioned if alarm calls are a form of altruism or nepotism in relation to relatives [87]. Here we assumed that during handling squirrels produced alarm calls, because these can serve typically as a warning call [53]. However, a call-type may also have more functions [51], e.g. being a mixed alarm and discomfort call.

## Conclusion

To sum up, in our study, the two squirrel populations affected by different level of human disturbance differed only in terms of their breath rate. This suggests, that squirrels in these two populations did not differ in medium-term and long-term stress in general, but they can differ in acute response to handling. No differences in the medium-term and long-term stress (i.e. cortisol and cortisone concentrations) between study sites suggests that both populations are similarly affected by environmental factors (e.g. season or weather conditions). The lack of clear effects, in this case, may also suggest that the populations are heterogeneous, which was shown in the body condition of individuals and breeding activity, specifically in the urban park population [14]. Indeed, it is thought that no single specific behavioral phenotype is favored by selection, because unpredictable and changing environmental conditions will favor different phenotypes and heterogeneous populations allow for adjustment to variable selective forces [58]. The varied response to human-induced stressors (e.g., handling) was likely due to the limited contact of forest squirrels with humans, whereas urban park squirrels have adapted to this ongoing anthropogenic stressor. This also indicates that breath rate was the best indicator of the acute stress response to handling [46]. This was confirmed by the higher breath rate in first-trapped squirrels. The indicators of acute stress, such as struggle rate and vocalization, appear to be less straightforward, as they are likely influenced by other factors that might not always be easy to interpret. For instance, it seems that the struggle rate was affected by environmental conditions (seasonal effects), and/or elevated glucocorticoids which have also been shown to influence behaviors [88]. Additionally, vocalization was probably intensified by the initial interaction with a human (handling), but its elevated level was only evident during the subsequent encounter. The summary above clearly demonstrates that in order to accurately assess the effects of anthropogenic stressors, it is advisable to employ a broader range of indicators and diverse analytical methods, including behavioral analyses. This approach enables a comprehensive and holistic examination of the matter at hand, as well as the identification of indicators suitable for future studies on the impact of urbanization on wildlife.

## Supporting information

S1 Table Average (±SD) values of indicators calculated from raw data for given groups with regard to SITE. (DOCX)

S2 Table Ranking of the models (ten highest ranked models and null model) explaining the long-term and medium-term stress in squirrels in generalized linear mixed models with gamma distribution and log link function (ΔAICc—AICc differences, ωi—Akaike weights, Rank—rank of the models based on AICc values; bolded text in the row indicates chosen model (for variable explanation, see: Methods). (DOCX)

S3 Table Indicator values (marginal averages), which are presented in Figs 2–7. (DOCX)

S4 Table Ranking of the models (ten highest ranked models and null model) explaining the acute stress in squirrels in generalized linear mixed models with gamma or negative binomial distribution and log link function (ΔAICc—AICc differences, ωi—Akaike weights, Rank—rank of the models based on AICc values; bolded text in the row indicates chosen model (for variable explanation, see: Methods). (DOCX)
