# Supplementary material for: Long-term, medium-term and acute stress response of urban populations of Eurasian red squirrels affected by different levels of human disturbance
Source: PLoS One. 2024 May 3;19(5):e0302933. doi: 10.1371/journal.pone.0302933 (PMC11068185; doi:10.1371/journal.pone.0302933)
Supplement: S1 Table — (DOCX) [file pone.0302933.s001.docx]

**Table S11.** Average (±SD) values of indicators calculated from raw data for given groups with regard to SITE.

|  | *URBAN FOREST* | | *URBAN PARK* | |
| --- | --- | --- | --- | --- |
|  | *Average* | *Standard Deviation* | *Average* | *Standard Deviation* |
| *Hair cortisol concentration [ug/g]* | | |  |  |
| *SEASON* |  |  |  |  |
| SPRING | 0.039 | 0.037 | 0.063 | 0.045 |
| SUMMER | 0.028 | 0.026 | 0.017 | 0.024 |
| AUTUMN | 0.027 | 0.011 | 0.030 | 0.028 |
| *EXPERIENCE* |  |  |  |  |
| FIRST-TRAPPED | 0.032 | 0.034 | 0.039 | 0.040 |
| RETRAPPED | 0.033 | 0.026 | 0.051 | 0.044 |
| *REPRODUCTIVE STATUS* |  |  |  |  |
| FEMALE BREEDING | 0.050 | 0.048 | 0.051 | 0.031 |
| FEMALE NONBREEDING | 0.024 | 0.019 | 0.035 | 0.033 |
| MALE BREEDING | 0.038 | 0.034 | 0.079 | 0.062 |
| MALE NONBREEDING | 0.029 | 0.022 | 0.034 | 0.030 |
| *Hair cortisone concentration [ug/g]*  *SEASON* |  |  |  |  |
| SPRING | 0.039 | 0.010 | 0.048 | 0.015 |
| SUMMER | 0.043 | 0.017 | 0.039 | 0.017 |
| AUTUMN | 0.058 | 0.013 | 0.055 | 0.022 |
| *EXPERIENCE* |  |  |  |  |
| FIRST-TRAPPED | 0.047 | 0.019 | 0.052 | 0.019 |
| RETRAPPED | 0.045 | 0.013 | 0.046 | 0.017 |
| *REPRODUCTIVE STATUS* |  |  |  |  |
| FEMALE BREEDING | 0.040 | 0.011 | 0.046 | 0.013 |
| FEMALE NONBREEDING | 0.053 | 0.018 | 0.055 | 0.021 |
| MALE BREEDING | 0.038 | 0.011 | 0.053 | 0.017 |
| MALE NONBREEDING | 0.048 | 0.015 | 0.040 | 0.013 |
| *Fecal cortisol concentration [ng/g]* | | |  |  |
| *SEASON* |  |  |  |  |
| SPRING | 37.60 | 41.19 | 84.09 | 80.32 |
| SUMMER | 20.46 | 14.79 | 16.22 | 12.18 |
| AUTUMN | 32.10 | 27.19 | 51.39 | 59.54 |
| WINTER |  |  | 90.33 | 93.16 |
| *EXPERIENCE* |  |  |  |  |
| FIRST-TRAPPED | 17.38 | 18.01 | 93.10 | 103.45 |
| RETRAPPED | 35.38 | 29.46 | 55.07 | 63.55 |
| *REPRODUCTIVE STATUS* |  |  |  |  |
| FEMALE BREEDING | 39.84 | 23.39 | 69.99 | 74.84 |
| FEMALE NONBREEDING | 26.11 | 40.14 | 81.41 | 89.29 |
| MALE BREEDING | 31.93 | 28.92 | 57.42 | 69.38 |
| MALE NONBREEDING | 25.24 | 16.89 | 36.81 | 51.81 |
| *Breath rate [chest moves/20 sec.]* | | |  |  |
| *SEASON* |  |  |  |  |
| SPRING | 29.621 | 5.031 | 26.135 | 3.742 |
| SUMMER | 26.400 | 3.069 | 27.636 | 4.756 |
| AUTUMN | 29.674 | 4.888 | 25.596 | 3.527 |
| WINTER | 28.300 | 4.547 | 24.226 | 2.880 |
| *EXPERIENCE* |  |  |  |  |
| FIRST-TRAPPED | 30.786 | 5.094 | 26.354 | 3.873 |
| RETRAPPED | 27.952 | 4.290 | 25.151 | 3.503 |
| *REPRODUCTIVE STATUS* |  |  |  |  |
| FEMALE BREEDING | 27.375 | 3.777 | 26.027 | 3.069 |
| FEMALE NONBREEDING | 28.838 | 4.330 | 25.808 | 3.879 |
| MALE BREEDING | 28.867 | 3.662 | 24.513 | 3.634 |
| MALE NONBREEDING | 28.673 | 5.276 | 25.750 | 3.756 |
| *Struggle rate [body moving time/30 sec.]* |  |  |  |  |
| *SEASON* |  |  |  |  |
| SPRING | 6.110 | 4.814 | 7.519 | 5.159 |
| SUMMER | 5.216 | 3.443 | 6.785 | 4.151 |
| AUTUMN | 8.272 | 5.698 | 7.073 | 6.217 |
| WINTER | 11.46 | 4.487 | 10.45 | 6.334 |
| *EXPERIENCE* |  |  |  |  |
| FIRST-TRAPPED | 8.960 | 4.112 | 8.573 | 5.701 |
| RETRAPPED | 6.598 | 5.319 | 7.595 | 6.097 |
| *REPRODUCTIVE STATUS* |  |  |  |  |
| FEMALE BREEDING | 7.598 | 3.437 | 7.544 | 5.851 |
| FEMALE NONBREEDING | 7.593 | 5.245 | 7.086 | 5.415 |
| MALE BREEDING | 8.941 | 4.425 | 9.727 | 7.288 |
| MALE NONBREEDING | 6.365 | 5.390 | 8.047 | 5.626 |
| *Vocalization [rank: 1-4]* |  |  |  |  |
| *SEASON* |  |  |  |  |
| SPRING | 1.667 | 0.959 | 1.593 | 1.002 |
| SUMMER | 1.806 | 1.046 | 2.043 | 1.186 |
| AUTUMN | 1.548 | 0.803 | 1.936 | 1.171 |
| WINTER | 1.200 | 0.632 | 1.761 | 1.139 |
| *EXPERIENCE* |  |  |  |  |
| FIRST-TRAPPED | 1.500 | 0.793 | 1.573 | 0.999 |
| RETRAPPED | 1.659 | 0.946 | 1.985 | 1.181 |
| *REPRODUCTIVE STATUS* |  |  |  |  |
| FEMALE BREEDING | 1.500 | 0.756 | 2.139 | 1.125 |
| FEMALE NONBREEDING | 1.757 | 1.038 | 1.867 | 1.256 |
| MALE BREEDING | 2.077 | 1.038 | 1.765 | 1.046 |
| MALE NONBREEDING | 1.436 | 0.764 | 1.649 | 1.010 |
