# Supplementary material for: Long-term, medium-term and acute stress response of urban populations of Eurasian red squirrels affected by different levels of human disturbance
Source: PLoS One. 2024 May 3;19(5):e0302933. doi: 10.1371/journal.pone.0302933 (PMC11068185; doi:10.1371/journal.pone.0302933)
Supplement: S2 Table — (DOCX) [file pone.0302933.s002.docx]

**Table S1.** Ranking of the models (ten highest ranked models and null model) explaining the long-term and medium-term stress in squirrels in generalized linear mixed models with gamma distribution and log link function (ΔAICc - AICc differences, ω_i_ - Akaike weights, Rank - rank of the models based on AICc values; bolded text in the row indicates chosen model (for variable explanation, see: methods).

| *Models* | *ΔAICc* | *ω_i_* | *Rank* |
| --- | --- | --- | --- |
| *Hair cortisol concentration* |  |  |  |
| **SEASON + CONDITION + AGE** | **0.0** | **0.155** | **1** |
| SITE + SEASON + CONDITION + AGE | 0.5 | 0.120 | 2 |
| SEASON + CONDITION + AGE + EXPERIENCE | 1.1 | 0.089 | 3 |
| SITE + SEASON + CONDITION + AGE + EXPERIENCE | 1.4 | 0.077 | 4 |
| SEASON + CONDITION | 2.0 | 0.057 | 5 |
| SITE + CONDITION + AGE + REPRODUCTIVE ST. | 2.3 | 0.049 | 6 |
| SEASON + CONDITION + AGE + REPRODUCTIVE ST. | 2.4 | 0.047 | 7 |
| SEASON + AGE | 3.3 | 0.030 | 8 |
| SITE + CONDITION + AGE + EXPERIENCE + REPRODUCTIVE ST. | 3.3 | 0.030 | 9 |
| SEASON + CONDITION + EXPERIENCE | 3.4 | 0.030 | 10 |
| … |  |  |  |
| *null model* | 20.5 | 0.000 | 64 |
| *Hair cortisone concentration* |  |  |  |
| **SEASON + CONDITION** | **0.0** | **0.214** | **1** |
| SEASON | 1.0 | 0.130 | 2 |
| SEASON + CONDITION + AGE | 1.9 | 0.083 | 3 |
| SITE + SEASON + CONDITION | 2.6 | 0.058 | 4 |
| SEASON + CONDITION + EXPERIENCE | 2.7 | 0.055 | 5 |
| SITE + SEASON | 3.4 | 0.039 | 6 |
| SEASON + EXPERIENCE | 3.6 | 0.035 | 7 |
| SEASON + AGE | 3.7 | 0.034 | 8 |
| CONDITION | 4.0 | 0.029 | 9 |
| SEASON + CONDITION + EXPERIENCE + AGE | 4.4 | 0.030 | 10 |
| … |  |  |  |
| *null model* | 5.1 | 0.017 | 14 |
| *Faecal cortisol concentration* |  |  |  |
| **SEASON + CONDITION** | **0.0** | **0.119** | **1** |
| SITE + SEASON + CONDITION | 0.1 | 0.114 | 2 |
| SEASON + CONDITION + AGE | 0.3 | 0.103 | 3 |
| SITE + SEASON + CONDITION + AGE | 0.5 | 0.093 | 4 |
| SEASON + CONDITION + EXPERIENCE | 1.2 | 0.066 | 5 |
| SITE + SEASON + CONDITION + EXPERIENCE | 1.3 | 0.062 | 6 |
| SEASON + CONDITION + EXPERIENCE + AGE | 1.5 | 0.056 | 7 |
| SITE + SEASON + CONDITION + EXPERIENCE + AGE | 1.6 | 0.054 | 8 |
| SEASON + CONDITION + REPRODUCTIVE ST. | 2.8 | 0.029 | 9 |
| SEASON | 3.0 | 0.027 | 10 |
| … |  |  |  |
| *null model* | 18.6 | 0.000 | 57 |
