# Supplementary material for: Long-term, medium-term and acute stress response of urban populations of Eurasian red squirrels affected by different levels of human disturbance
Source: PLoS One. 2024 May 3;19(5):e0302933. doi: 10.1371/journal.pone.0302933 (PMC11068185; doi:10.1371/journal.pone.0302933)
Supplement: S3 Table — (DOCX) [file pone.0302933.s003.docx]

**Table S3.** Indicator values (marginal averages), which are presented in figures 2-7.

| *Figure 2 - Mean (±SE) hair cortisol concentration in squirrels with regard to SEASON (marginal means from generalized linear mixed model)* *[ug/g]* | | |
| --- | --- | --- |
| *SEASON* | *Marginal mean* | *Standard Error* |
| SPRING | 0.03 | 0.03 |
| SUMMER | 0.01 | 0.01 |
| AUTUMN | 0.02 | 0.02 |
| *Figure 3 - Mean (±SE) hair cortisone concentration in squirrels with regard to SEASON (marginal means from generalized linear mixed model)* *[ug/g]* | | |
| *SEASON* | *Marginal mean* | *Standard Error* |
| SPRING | 0.046 | 0.012 |
| SUMMER | 0.041 | 0.012 |
| AUTUMN | 0.057 | 0.012 |
| *Figure 4 - Mean (±SE) fecal cortisol concentration in squirrels with regard to SEASON (marginal means from generalized linear mixed model)* *[ng/g]* | | |
| *SEASON* | *Marginal mean* | *Standard Error* |
| SPRING | 30.4 | 26.4 |
| SUMMER | 16.6 | 14.7 |
| AUTUMN | 32.8 | 29.4 |
| WINTER | 58.6 | 51.5 |
| *Figure 5. Mean (±SE) breath rate in squirrels with regard to A) EXPERIENCE (first-trapped or retrapped) and B) SITE (urban park or urban forest) (marginal means from generalized linear mixed model). [chest moves/20 sec.]* | | |
| *EXPERIENCE* | *Marginal mean* | *Standard Error* |
| FIRST-TRAPPED | 28.3 | 2.9 |
| RE-TRAPPED | 26.5 | 2.7 |
| *SITE* | *Marginal mean* | *Standard Error* |
| URBAN PARK | 25.9 | 2.7 |
| URBAN FOREST | 28.9 | 3.0 |
| *Figure 6. Mean (±SE) struggle rate in squirrels with regard to SEASON (marginal means from generalized linear mixed model). [body moving time/30 sec.]* | | |
| *SEASON* | *Marginal mean* | *Standard Error* |
| SPRING | 8.0 | 4.8 |
| SUMMER | 6.9 | 4.2 |
| AUTUMN | 7.7 | 4.7 |
| WINTER | 11.2 | 6.8 |
| *Figure 7. Mean (±SE) vocalization in squirrels with regard to EXPERIENCE (first-trapped or retrapped) (marginal means from generalized linear mixed model). [rank: 1-4]* | | |
| *EXPERIENCE* | *Marginal mean* | *Standard Error* |
| FIRST-TRAPPED | 1.5 | 0.6 |
| RE-TRAPPED | 1.9 | 0.7 |
