# Supplementary material for: Long-term, medium-term and acute stress response of urban populations of Eurasian red squirrels affected by different levels of human disturbance
Source: PLoS One. 2024 May 3;19(5):e0302933. doi: 10.1371/journal.pone.0302933 (PMC11068185; doi:10.1371/journal.pone.0302933)
Supplement: S4 Table — (DOCX) [file pone.0302933.s004.docx]

**Table S3.** Ranking of the models (ten highest ranked models and null model) explaining the acute stress in squirrels in generalized linear mixed models with gamma or negative binomial distribution and log link function (ΔAICc - AICc differences, ω_i_ - Akaike weights, Rank - rank of the models based on AICc values; bolded text in the row indicates chosen model (for variable explanation, see: methods).

| *Models* | *ΔAICc* | *ω_i_* | *Rank* |
| --- | --- | --- | --- |
| *Breath rate* |  |  |  |
| **SITE + CONDITION + EXPERIENCE** | **0.0** | **0.465** | **1** |
| SITE + EXPERIENCE | 1.3 | 0.243 | 2 |
| SITE + CONDITION | 3.4 | 0.085 | 3 |
| SITE | 3.9 | 0.066 | 4 |
| SITE + CONDITION + AGE + EXPERIENCE | 3.9 | 0.066 | 5 |
| SITE + AGE + EXPERIENCE | 4.6 | 0.047 | 6 |
| SITE + CONDITION + AGE | 7.4 | 0.011 | 7 |
| SITE + AGE | 7.6 | 0.010 | 8 |
| SITE + SEASON + CONDITION + EXPERIENCE | 10.3 | 0.003 | 9 |
| SITE + SEASON + EXPERIENCE | 11.3 | 0.002 | 10 |
| … |  |  |  |
| *null model* | 22.2 | 0.000 | 28 |
| *Struggle rate* |  |  |  |
| **SEASON + CONDITION** | **0.0** | **0.257** | **1** |
| SEASON + CONDITION + EXPERIENCE | 1.6 | 0.116 | 2 |
| SEASON | 2.7 | 0.067 | 3 |
| SEASON + CONDITION + AGE | 2.7 | 0.067 | 4 |
| SITE + SEASON + CONDITION | 2.8 | 0.063 | 5 |
| CONDITION | 3.6 | 0.043 | 6 |
| SEASON + CONDITION + EXPERIENCE + AGE | 4.1 | 0.033 | 7 |
| SITE + SEASON + CONDITION + EXPERIENCE | 4.4 | 0.028 | 8 |
| SITE + CONDITION | 4.5 | 0.027 | 9 |
| SEASON + CONDITION + REPRODUCTIVE ST. | 4.8 | 0.023 | 10 |
| … |  |  |  |
| *null model* | 6.0 | 0.013 | 18 |
| *Vocalization* |  |  |  |
| **CONDITION + EXPERIENCE** | **0.0** | **0.172** | **1** |
| CONDITION | 0.3 | 0.148 | 2 |
| CONDITION + EXPERIENCE + AGE | 1.2 | 0.094 | 3 |
| CONDITION + AGE | 1.2 | 0.094 | 4 |
| SITE + CONDITION + EXPERIENCE | 1.6 | 0.077 | 5 |
| SITE + CONDITION | 2.2 | 0.057 | 6 |
| *null model* | 2.6 | 0.047 | 7 |
| SITE + CONDITION + EXPERIENCE + AGE | 2.8 | 0.042 | 8 |
| SITE + CONDITION + AGE | 3.2 | 0.035 | 9 |
| EXPERIENCE | 3.2 | 0.035 | 10 |
